# DML–LLM Hybrid Architecture for Fault Detection and Diagnosis in Sensor-Rich Industrial Systems

**DOI:** 10.3390/s26062008

**Published:** 2026-03-23

**Authors:** Yu-Shu Hu, Saman Marandi, Mohammad Modarres

**Affiliations:** 1 DML Inc., Chubei, Hsinchu 30274, Taiwan; 2 Center for Risk and Reliability, A.J. Clark School of Engineering, University of Maryland, College Park, MD 20742, USA; smarandi@umd.edu (S.M.); modarres@umd.edu (M.M.)

**Keywords:** large language models, diagnostics, reasoning, dynamic master logic (DML), fault detection and diagnosis (FDD), causal inference, temporal fuzzy logic, soft thresholds, neuro-symbolic hybrid, state-aware orchestration

## Abstract

Fault Detection and Diagnosis (FDD) in complex industrial systems requires methods that can handle uncertain operating conditions, soft thresholds, evolving sensor behavior, and increasing volumes of heterogeneous data. Traditional model-based or rule-driven approaches offer interpretability but lack adaptability, while purely data-driven and Large Language Model (LLM)-based methods often struggle with consistency, traceability, and causal grounding. Dynamic Master Logic (DML) provides a causal and temporal reasoning structure with fuzzy rules that capture gradual drift, soft limits, and asynchronous sensor signals while preserving traceability and deterministic evidence propagation. Building on this foundation, this paper presents a DML–LLM hybrid architecture that integrates targeted LLM inference to interpret unstructured information such as logs, notes, or retrieved documents under controlled prompts that maintain domain constraints. The combined system integrates Bayesian updating, deterministic routing, and semantic interpretation into a unified FDD pipeline. In a semiconductor manufacturing case study, the proposed framework reduced time to detection (TTD) from 7.4 h to 1.2 h and improved the F1 score from 0.59 to 0.83 when compared with conventional Statistical Process Control (SPC) and Fault Detection and Classification (FDC) workflows. Provenance completeness increased from 18% to 96%, while engineer triage time was reduced from 72 min to 18 min per event. These results demonstrate that the hybrid framework provides a scalable and explainable approach to anomaly detection and fault diagnosis in sensor-rich industrial environments.

## 1. Introduction

Fault Detection and Diagnosis (FDD) is crucial for maintaining safety and reliability in complex industrial systems such as aerospace, semiconductor manufacturing, and energy infrastructure. Traditional FDD approaches can be broadly categorized into model-based (physics or simulation models), knowledge-based (expert rules, fault trees), and data-driven techniques (signal processing, statistical analysis, machine learning) [1]. Model-based and expert-driven methods offer interpretability and clear causal rationale, but they rely on extensive domain knowledge and often use rigid threshold rules that struggle with uncertainty and evolving system behaviors. In contrast, data-driven approaches (from statistical monitoring to deep learning) can automatically detect anomalies in large multivariate data but tend to behave as “black boxes,” lacking transparency in reasoning [2].

Data-driven FDD methods leverage machine learning and statistical analysis on historical sensor data to automatically learn fault patterns. Such methods can detect subtle anomalies in high-dimensional data and handle nonlinear relationships without extensive prior modeling [3]. Indeed, the integration of Artificial Intelligence (AI) has led to measurable improvements in anomaly detection and predictive maintenance in industry. Despite these advantages, data-driven models face critical challenges. They require large quantities of labeled fault data for training, and their accuracy and stability can degrade when real operating conditions deviate from the training distribution. In industrial practice, novel faults and non-stationary conditions (e.g., changing loads, environmental drift, aging) are inevitable, and a trained model may not recognize a fault it has never seen or may misclassify conditions under shifting data patterns [4,5].

Recent research has thus gravitated toward hybrid architectures that combine logical reasoning with advanced AI-driven analytics. Large Language Models (LLMs) have emerged as powerful tools to interpret and generate human-like explanations from unstructured data. Pre-trained LLMs can ingest sensor descriptions, maintenance logs, and operating manuals, offering a natural language interface to industrial data and leveraging vast general knowledge to hypothesize causes and suggesting remedies [6,7]. When augmented with the retrieval of relevant domain documents, LLMs are capable of contextual reasoning [8]. These abilities make LLMs attractive for FDD, enabling flexible integration of textual and visual information and interactive question-answering for operators.

However, purely generative AI approaches on their own lack the grounded, traceable reasoning required in safety-critical settings. They often cannot inherently represent the causal structure of physical processes or guarantee consistent application of domain constraints. In general, LLMs may hallucinate plausible sounding but incorrect explanations, and their conclusions are not easily auditable step by step [9]. In short, while LLMs provide remarkable language understanding and pattern recognition, they do so as opaque inference engines, with limited interpretability or compliance assurance by default.

Dynamic Master Logic (DML) offers a complementary solution by serving as a symbolic and causal model for diagnostics. DML is a formal modeling framework that encodes expert knowledge, including procedures, dependencies, and heuristics, into a hierarchical logic diagram that is often represented as a graph of goals, events, and functions [10]. It was originally developed to capture complex system behavior in a compact, interpretable structure, and has since been applied to reliability and risk analysis in domains like nuclear safety [11]. Unlike statistical black-box models, DML explicitly represents temporal and causal relationships (e.g., sequences of events, if-then rules with fuzzy thresholds for gradual degradations), enabling end-to-end traceability from symptoms to root causes.

Every decision in a DML-based diagnosis follows a rule-based path that can be inspected and audited by engineers or regulators. This inherent transparency and the ability to handle uncertainty (via fuzzy logic and graded truth values) directly address the shortcomings of traditional FDD methods in dealing with dynamic, uncertain conditions. In essence, DML provides the interpretable “control-logic” foundation on which reliable diagnostic reasoning can be built.

Beyond its role as an explicit diagnostic logic backbone, DML is inherently designed to evolve with accumulating operational data. As real-time field data streams are continuously ingested, DML can update belief states and rule parameters through fuzzy statistical updating or Bayesian inference, enabling existing rules to be recalibrated and context-specific diagnostic routes to be instantiated without changing the underlying causal topology. In this manner, expert knowledge encoded in DML is not static, but progressively calibrated to reflect changing system behavior, uncertainty, and gradual degradation phenomena.

Crucially, incoming data streams are transformed into fuzzy sets prior to inference, enabling DML to reason over graded states rather than binary thresholds. These fuzzy representations modulate the activation of goals, events, and functions within the DML hierarchy, which in turn governs the invocation of different LLM prompts and agent workflows. As system states evolve, distinct fuzzy activation patterns trigger context-specific prompts, analytical roles, or reasoning agents, ensuring that LLM interactions remain tightly coupled to the current diagnostic context.

This mechanism directly addresses a fundamental limitation of standalone LLM-based approaches: the lack of intrinsic dynamic interaction and state awareness. Rather than relying on a monolithic prompt or static conversational flow, the proposed architecture uses DML as a stateful control layer that orchestrates when, how, and for what purpose LLM reasoning is invoked. Consequently, LLMs operate as adaptive reasoning modules embedded within a continuously updated causal framework, rather than as isolated generative engines. This DML-guided, data-driven interaction paradigm enables responsive, traceable, and context-aware diagnostic reasoning suitable for dynamic and safety-critical industrial environments.

Overall, the integration of DML and LLM unites the transparent, rule-based reasoning of expert systems with the adaptability of generative AI, supporting accountable and intelligent fault management suited for safety-critical environments. Based on the challenges identified in complex industrial diagnostic environments, the primary contributions of this research to the field of fault detection and diagnosis (FDD) in sensor-rich systems are threefold:

A Causal–Generative Hybrid FDD Architecture: We propose a novel framework that integrates DML as the primary diagnostic reasoning backbone. By utilizing LLMs strictly as constrained auxiliary interpreters for unstructured information, the architecture ensures causal traceability and auditability—qualities that are fundamentally absent in standalone LLM-based approaches.

A State-Aware Orchestration Mechanism: We develop a governance layer in which DML governs the timing, scope, and role of LLM inference based on evolving fuzzy system states. This enables context-specific reasoning under non-stationary operating conditions without relinquishing deterministic control of the diagnostic workflow.

Empirical Validation via Industrial Case Study: Through a validated application in semiconductor manufacturing, we demonstrate that the proposed hybrid framework achieves earlier anomaly detection, improved diagnostic accuracy, and stronger provenance compared with conventional SPC/FDC methods and purely data-driven or LLM-centric pipelines.

The remainder of this paper is organized as follows: Section 2 provides background on FDD, DML, and LLMs. Section 3 presents the hybrid reasoning approach, while Section 4 describes the integrated framework execution flow. Section 5 introduces a semiconductor case study and quantitative results. Section 6 offers a discussion on limitations, and Section 7 concludes with implications for high-reliability industries.

## 2. Background

### 2.1. Overview of Data Evolution and Fault Detection and Diagnostics

The evolution of FDD has been shaped by advances in sensing, computation, and data management. It is more accurate to describe this development as a gradual expansion in data scale, frequency, and variety rather than a simple shift from low-dimensional sensor readings to multimodal data. Early industrial systems relied mainly on measurements collected at limited sampling rates due to hardware and storage constraints. Diagnostic methods were therefore centered on simplified feature extraction, periodic monitoring, and offline rule-based or model-based inference. These approaches provided interpretable results, although their adaptability to changing operating conditions was limited. Researchers have introduced robust and adaptive filtering methods, but their practical flexibility remained restricted by available computational resources [12,13].

Improvements in sensing technologies, networking infrastructure, and high-performance computing have gradually expanded both the volume and the diversity of data available. Modern industrial installations may incorporate high-resolution time-series measurements together with additional modalities such as imaging systems, machine vision outputs, maintenance logs, and textual operator records. The extent to which such multimodal data are available differs across industries and equipment types. This broader data landscape has encouraged the development of several methodological families within contemporary FDD research. Each family tends to be associated with particular data characteristics, although none is restricted to a single format [14,15].

Signal processing methods continue to play a central role in analyzing time-series measurements and vibration or process signals. Techniques such as spectral analysis, wavelet transforms, cepstral features, and statistical process control remain widely used for detecting anomalies and extracting discriminative patterns [16]. As system complexity and data volumes increased, these techniques were supplemented by large-scale data mining capable of discovering patterns within structured and semi-structured sources [17].

The expert knowledge-driven family built upon human reasoning to formalize causal and procedural relationships. Methods such as rule-based systems, Fault Tree Analysis [18], and Failure Mode and Effects Analysis (FMEA) [19] provided interpretable reasoning chains but required labor-intensive maintenance. Later frameworks introduced dynamic logic structures capable of adapting expert rules to evolving contexts while retaining auditability. Model-based FDD was developed in parallel by exploiting physical and analytical representations of system dynamics. Residual generation methods, Kalman filters, observers, and Bayesian classification techniques have enabled accurate state estimation and fault isolation when suitable system models exist. Their performance, however, depends strongly on model fidelity and on robustness to parameter variation. This limitation has motivated extensive research into robust, adaptive, and hybrid observers [20].

Recent computational advances have increased interest in hybrid and multimodal fault detection and diagnosis (FDD) approaches. These methods aim to combine the precision of signal-level analysis with the adaptability of data-driven techniques and the interpretability of knowledge-based reasoning. Examples include physics-informed machine learning [21], data-fusion frameworks [22], and diagnostic architectures capable of processing heterogeneous information such as sensor signals, images, and textual data within a unified workflow. Although fully unified cross-domain diagnostic systems remain an active research topic and are not yet widely deployed in typical industrial environments, current developments indicate a clear shift toward more integrated and context-aware diagnostic reasoning.

### 2.2. The Shift from Numerical to Multimodal Data in Modern FDD

Traditional FDD systems were primarily designed for numerical sensor measurements such as temperature, pressure, flow rate, and electrical signals. These scalar or low-dimensional variables were typically analyzed using threshold-based monitoring, statistical process control (SPC), or model-based residual analysis. While effective for many industrial applications, these approaches assume that system behavior can be adequately represented through a limited set of numerical process variables.

However, modern industrial environments—particularly advanced semiconductor fabrication facilities—generate far richer diagnostic information. Contemporary manufacturing systems produce heterogeneous and high-dimensional data streams including wafer inspection images, defect maps, equipment vibration signals, acoustic signatures, and other specialized sensing modalities. These observations capture spatial, temporal, and spectral characteristics of system behavior that cannot be directly observed through conventional scalar measurements.

The availability of multimodal sensing significantly improves fault observability and enables earlier detection of subtle process deviations. For example, spatial patterns in wafer inspection images or gradual shifts in vibration spectra may reveal early indications of tool degradation or process drift before they manifest in conventional process parameters.

Despite these advantages, integrating heterogeneous diagnostic evidence remains challenging. Multimodal data sources differ substantially in structure, dimensionality, and semantic meaning, making it difficult to combine them within traditional FDD frameworks. Numerical signals are typically processed using statistical or machine learning techniques, whereas images and spectral data require specialized computer vision or signal processing algorithms. Coordinating these heterogeneous analytical methods within a unified diagnostic workflow therefore presents a significant challenge.

Recent studies have explored artificial intelligence techniques—including deep learning and large language models (LLMs)—to interpret complex industrial data. While these approaches improve the ability to detect subtle anomalies in high-dimensional observations, many multimodal AI models operate as opaque inference systems with limited interpretability and weak causal grounding. Such characteristics can hinder traceable diagnostic explanations and reduce robustness under non-stationary operating conditions, which frequently arise in semiconductor manufacturing due to process drift, equipment aging, and frequent recipe updates.

Consequently, although multimodal sensing greatly enhances the observability of industrial systems, there remains a lack of diagnostic frameworks capable of integrating heterogeneous evidence while preserving explicit, auditable, and causally grounded reasoning processes. Addressing this gap motivates the structured reasoning architecture introduced in the following sections.

**Figure 1:**
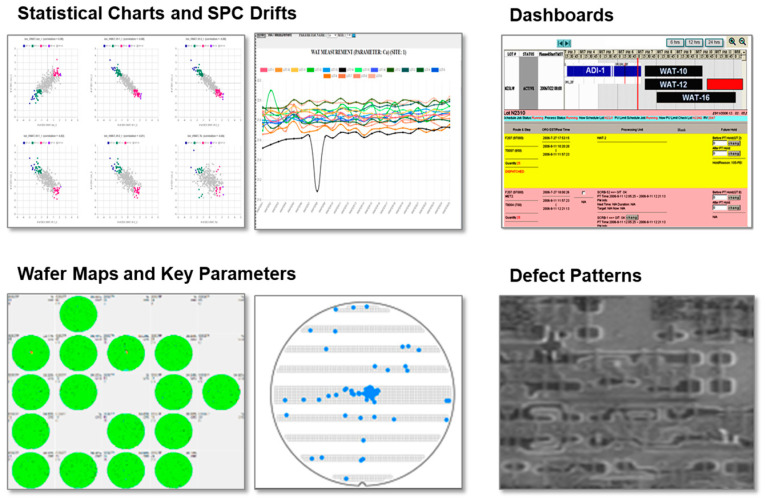
Representative multimodal diagnostic data in semiconductor manufacturing, including SPC/FDC parameter trends, Wafer Acceptance Test (WAT) summaries, wafer maps, defect spatial distributions, and inspection images, illustrating the heterogeneous evidence sources required for modern FDD.

In sensor-rich semiconductor manufacturing environments, fault diagnosis must therefore transition from purely numerical monitoring toward multimodal reasoning frameworks capable of integrating heterogeneous evidence within a unified diagnostic context, as illustrated in Figure 1.

An important enabling mechanism for multimodal reasoning is the use of fuzzy logic as an intermediate representation layer. Unlike conventional crisp thresholds, fuzzy logic employs linguistic variables (e.g., low, medium, high, increasing, unstable) and soft-threshold membership functions to represent system states in a graded and interpretable manner. This representation is particularly suitable for multimodal observations whose semantics cannot be captured by precise numerical boundaries.

By mapping heterogeneous features—including numerical signals, image-derived metrics, and spectral characteristics—into fuzzy sets, diverse data types can be normalized into a common semantic space while preserving uncertainty and gradual transitions. In this way, fuzzy logic provides a unifying abstraction layer between raw multimodal measurements and higher-level diagnostic reasoning. Table 1 This capability forms a natural bridge for integrating multimodal observations into structured reasoning frameworks such as Dynamic Master Logic (DML), enabling consistent interpretation across modalities while maintaining transparency and interpretability.

### 2.3. Dynamic Master Logic as a Reasoning Framework

**Figure 2:**
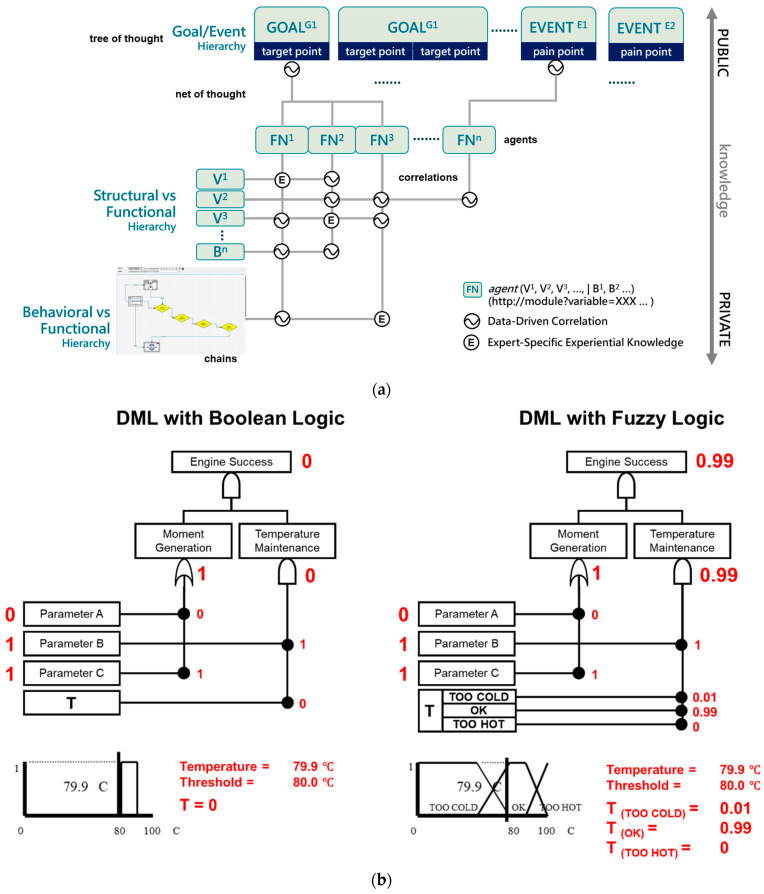

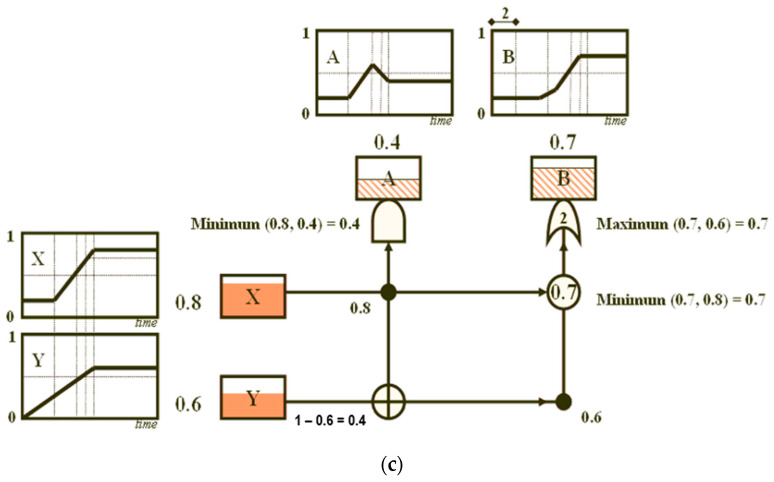
(**a**). DML decomposes complex systems into hierarchical goal, event, functional, structural, and behavioral layers. (**b**). Boolean logic vs. fuzzy logic in threshold reasoning. (**c**). Fuzzy logic enables more nuanced, time-aware system evaluations, preventing premature failure declarations based on near-miss inputs. In this case A = X ∩ not Y; B = (0.7X ∪ Y)^2^.

In many deployments, diagnostic success depends less on the sheer scale of language models and more on the ability to capture, organize, and operate fragmented and tacit expert knowledge. Dynamic Master Logic provides a formal control-logic framework that encodes procedures, constraints, and expert heuristics into auditable and executable structures. Initially proposed by Hu and Modarres [10,11,23] and later extended in Knowledge Graph–DML diagnostic frameworks [24], DML decomposes complex systems into a set of explicitly layered hierarchies, as illustrated in Figure 2a.

At the top of the hierarchy, goal layers represent system-level objectives or success criteria (target points), while event layers capture observable symptoms, anomalies, or pain points that indicate deviations from those goals. Beneath these, the functional layer describes what the system is intended to do, such as control, regulation, or transformation, linking high-level goals to realizable system capabilities. The structural layer represents the physical or logical components (for example, subsystems, modules, variables, or parameters) that implement these functions, while the behavioral layer encodes dynamic response patterns, operational sequences, and condition-dependent chains that describe how structures and functions evolve over time.

These layered hierarchies are not independent; rather, they are interconnected through explicit causal and temporal links that govern how evidence propagates across layers. As shown in Figure 2a, evidence originating at the structural or behavioral level (e.g., parameter deviations or dynamic chains) is routed upward through functional logic toward event and goal nodes, while high-level diagnostic hypotheses can also be traced downward to identify contributing components and behaviors. Temporal operators and fuzzy logic are embedded within these inter-layer connections, allowing DML to represent gradual degradation, partial satisfaction of conditions, and asynchronous effects instead of enforcing brittle binary transitions.

Each link between DML nodes is defined by subject-matter experts, who specify the strength and nature of influence between components using engineering knowledge, system design documentation, failure analyses, operational thresholds, and domain experience. These relationships are not learned statistically; instead, they form an explicit rule base that determines how diagnostic evidence is routed, weighted, and combined across the hierarchy. This expert-defined, multi-layer structure ensures that every dependency in the model is transparent, interpretable, and physically meaningful, enabling end-to-end traceability from low-level observations to high-level diagnostic conclusions and recommended actions.

Figure 2b contrasts crisp Boolean threshold logic with its fuzzy counterpart using the same physical temperature condition. In the Boolean implementation (left), system reasoning is governed by hard thresholds: when the measured temperature (79.9 °C) falls just below the predefined limit (80.0 °C), the temperature condition is evaluated as false (T = 0). This binary decision then propagates upward through the logic tree, potentially collapsing higher-level success evaluation despite a marginal deviation.

In contrast, the fuzzy implementation (right) replaces crisp pass/fail evaluation with graded membership functions (e.g., TOO COLD, OK, TOO HOT). Under the same temperature condition, the fuzzy representation assigns partial memberships (e.g., T(OK) = 0.99, T (TOO COLD) = 0.01), allowing the Temperature Maintenance node to remain largely satisfied rather than abruptly failing. These graded values propagate through fuzzy logic gates, resulting in a high overall Engine Success confidence (0.99) instead of a binary failure. By allowing overlapping states and smooth transitions, fuzzy logic captures uncertainty, sensor noise, and near-threshold behavior that Boolean logic cannot represent.

Within the DML framework, this distinction is critical: Boolean logic enforces brittle decision boundaries, whereas fuzzy logic enables proportional reasoning, temporal smoothing, and confidence-aware propagation. As a result, DML avoids false alarms caused by marginal threshold crossings and supports diagnostic conclusions that more faithfully reflect real physical system behavior under gradual drift and uncertainty. A key feature of DML is its treatment of time as a logical dimension. Faults and sensor signals rarely evolve synchronously, and DML captures such dynamics by introducing temporal operators that align delayed or asynchronous inputs. Through temporal fuzzy propagation, the framework evaluates how each signal evolves over time and limits its conclusions based on the confidence assigned to the corresponding relationships. This approach produces smooth transitions in reasoning rather than abrupt logical flips, yielding results that more accurately reflect real-world system behavior. Temporal reasoning also enables DML to distinguish transient anomalies from persistent trends, ensuring that diagnostic conclusions are informed by both the sequence and duration of observed events.

By combining fuzzy, temporal, and causal reasoning within a single interpretable model, DML provides a bridge between qualitative expert logic and quantitative data. The framework has proven effective in safety-critical diagnostics and control applications, where traceability and graded reasoning are required to manage uncertainty and evolving operational conditions. The mathematical formulation of temporal fuzzy propagation, including Degree of Failure Membership (DFM) operators and bounded logic gates, follows the original DML description provided in Hu and Modarres [10].

The output of the DML framework represents diagnostic inferences such as likely fault sources, contributing factors, and confidence levels, all derived through causal and temporal reasoning over system evidence. Each result is traceable, allowing engineers to follow how observations propagate through the model to produce high-level explanations or alerts, as shown in Figure 2c. Importantly, while the structural relationships in DML are expert-defined and fixed, belief values and confidence levels can be updated through fuzzy statistical or Bayesian mechanisms without altering the underlying causal topology.

### 2.4. Large Language Models for Flexible Knowledge Reasoning

Recent advances in sensing, storage, and computation have transformed how FDD systems operate. Industrial processes now generate continuous streams of multimodal data, including sensor measurements, maintenance logs, inspection images, and operator notes. This expansion has enabled a shift from purely numerical analysis to reasoning approaches that incorporate language, vision, and process semantics.

Large Language Models have emerged as powerful tools in this context, offering the ability to interpret, relate, and reason across diverse types of information. By analyzing process descriptions alongside sensor data and textual records, LLMs enable more flexible and context-aware diagnostic reasoning. This integration supports the alignment of human-centric knowledge with real-time operational evidence [5,7].

Despite these capabilities, LLMs face several important limitations. One major concern is hallucination, where the model generates information that appears plausible but is factually incorrect or fabricated. This occurs because LLMs rely on statistical patterns in their training data rather than fact verification. In addition, LLMs cannot access private or real-time information, as they are restricted to the static data available at the time of training and lack native mechanisms for external retrieval. They also struggle with complex reasoning tasks, particularly those requiring step-by-step logic, causal relationships, or temporal consistency. These challenges limit their effectiveness in applications that demand high accuracy and domain specific control [25,26].

To overcome the inherent limitations of standalone LLMs, hybrid approaches have been introduced. These include frameworks that combine LLMs with external knowledge sources, such as retrieval-augmented generation (RAG) pipelines for unstructured text retrieval, and knowledge graphs [27] for structured, entity-based reasoning [28]. These systems enhance factual grounding and traceability by conditioning LLM outputs on external data retrieved from curated knowledge bases. Architectures like Dense Passage Retrieval (DPR) [29] and Fusion-in-Decoder (FiD) [30] exemplify this trend, enabling LLMs to draw from verified sources during inference. Additionally, emerging governance frameworks like the Model Context Protocol (MCP) [31] support access control, data integrity, and auditability across hybrid systems.

In parallel, a second class of innovations focuses on improving the internal reasoning capabilities of language models. Techniques such as Mixture-of-Experts (MoE) [32], agentic workflows such as ReAct [33], Toolformer [34], and reasoning methods like Chain of Thought (CoT) [35], Tree of Thoughts (ToT) [36] of Self Consistency [37] prompting have expanded the expressiveness of LLMs. These enhancements improve a model’s ability to plan, revise, and explain its reasoning steps. However, even with these improvements, LLMs continue to face challenges in domains that demand traceability, temporal consistency, and compliance with domain constraints.

**Figure 3:**
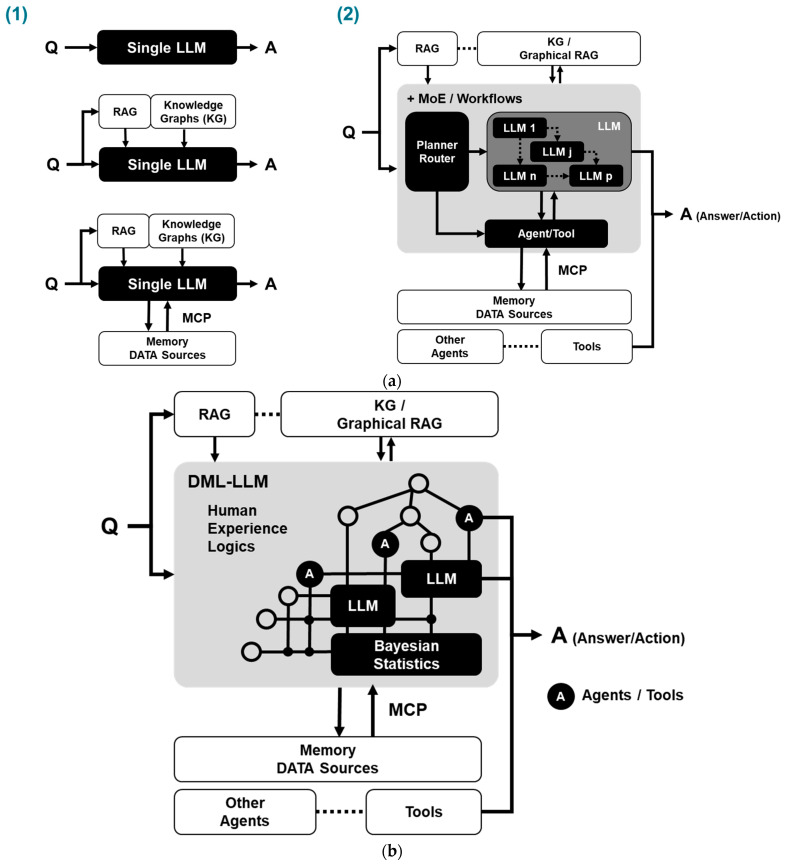

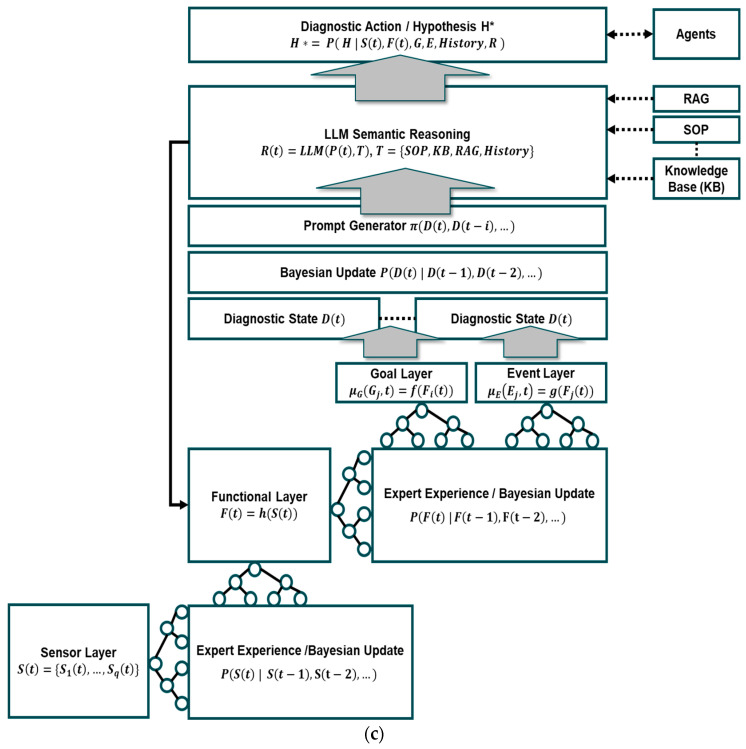
(**a**). The first path (**1**) enriches LLMs through external knowledge integration, while the second path (**2**) improves internal orchestration and reasoning structure. (**b**). Proposed integrated DML–LLM architecture. (**c**). Hybrid DML–LLM diagnostic architecture where sensor observations are transformed into functional states, forming the diagnostic state $D_{t}$, which is updated through Bayesian inference and interpreted by LLMs to generate diagnostic decisions.

Figure 3a illustrates these two complementary directions. The first path, as shown in Figure 3a(1), improves LLMs through external knowledge integration, while the second path, as shown in Figure 3a(2), improves internal orchestration and reasoning structure. Together, these developments support more adaptive diagnostic workflows, yet they do not fully resolve the need for transparent and auditable reasoning in safety-critical environments. In many industrial settings, key causal or contextual relationships remain uncertain or incomplete, and existing diagnostic models cannot always represent them. When such gaps arise, external sources such as technician notes, maintenance reports, and retrieved documents provide essential information that must be interpreted in context. LLMs are well-suited to extract meaning from this external evidence and supply the missing semantic relationships, while DML provides the causal and temporal backbone needed to evaluate these signals reliably. This motivates the hybrid reasoning approach described in the next section.

However, these approaches alone do not define how such hybrid reasoning should be operationalized within a state-aware diagnostic workflow, which is the focus of the proposed architecture in Section 3.

Because prior studies span heterogeneous industrial domains and evaluation protocols, direct quantitative comparison across works is not methodologically consistent; therefore, this study focuses on controlled baseline comparisons within a unified evaluation framework.

Recent neuro-symbolic reasoning frameworks integrate symbolic logic with neural models to improve explainability. However, most approaches rely on static rule structures or graph-based reasoning. In contrast, the proposed DML–LLM framework introduces a dynamic causal–temporal reasoning backbone where belief propagation is governed by fuzzy logic and Bayesian updating.

Unlike typical neuro-symbolic systems that combine neural models with symbolic reasoning at the representation level, the proposed architecture positions DML as the primary causal–temporal reasoning backbone while LLMs operate only as constrained semantic interpreters for unstructured information.

## 3. Research Overview: Toward Hybrid Reasoning

This section outlines how the proposed hybrid framework integrates DML and LLM components into a single diagnostic workflow for FDD. The workflow begins by processing evidence from structured and unstructured sources, including SPC and FDC signals, drift patterns, metrology deviations, and retrieved contextual notes. DML evaluates these inputs using fuzzy membership functions and temporal operators, which quantify deviation levels and determine whether conditions persist across required time windows or align with expected event sequences. Bayesian estimators update belief values under uncertainty, and the DML structure specifies how these values propagate through the causal and temporal pathways.

LLMs are incorporated only when the system encounters information that cannot be interpreted through DML rules alone. In these cases, DML generates a logic-guided prompt containing the node context, the missing information, and the constraints that the LLM must follow. This ensures that the model performs a targeted interpretation task rather than open-ended inference. Typical tasks include extracting meaning from technician notes, identifying relationships described in maintenance reports, or providing a semantic summary of relevant domain documents.

Any LLM output is returned as a controlled inference signal, which is then normalized through deterministic functions within DML. These functions apply stability checks, weighting rules, thresholds, or fuzzy logic to maintain consistency with the causal and temporal structure. If the output is unstable or inconsistent with domain logic, its influence is reduced or discarded. This integration approach allows the system to benefit from flexible semantic interpretation while ensuring that all reasoning remains explicit, governed by traceable rules, and consistent with physical and operational constraints.

The combined architecture supports both forward reasoning, which evaluates possible downstream effects of a hypothesized cause, and backward reasoning, which identifies potential contributors to an observed symptom. When data alone cannot fully explain a pattern, DML may request a focused LLM interpretation of unstructured signals. All inference steps are logged, allowing the complete reasoning chain to be reviewed and audited. Figure 3b illustrates this integration, showing how DML provides the causal–temporal reasoning backbone while LLM modules, Bayesian statistics, RAG/KG retrieval, and MCP-managed data sources interact to form a unified diagnostic loop from query (Q) to answer or action (A).

Unlike loosely coupled hybrid architectures, the proposed framework positions DML as the primary logical control layer, with LLMs operating strictly as subordinate, task-specific inference modules.

### 3.1. Conceptual Architecture of the DML–LLM Hybrid Framework

The DML–LLM hybrid architecture integrates symbolic reasoning, probabilistic updating, and semantic interpretation within a unified diagnostic framework. DML provides the causal–temporal reasoning backbone, Bayesian inference captures dynamic credibility of relationships, and LLMs provide contextual interpretation of unstructured knowledge sources. The reasoning space is defined using goal and event structures.

Goal set:

$$G\;=\;\{G_{1},\;G_{2},\;\ldots \;,\;G_{n}\}$$

Event set:

$$E\;=\;\{E_{1},\;E_{2},\;\ldots \;,\;E_{k}\}$$

Diagnostic reasoning space:

Ω = G ∪ E

Goals represent desired system objectives, while events represent undesirable conditions or risks that must be detected and mitigated. An illustrative example of the goal–event modeling procedure is provided in Appendix A. The appendix demonstrates how system objectives (Goals) and undesirable operational conditions (Events) are systematically identified from engineering knowledge, sensor observations, and historical diagnostic reports. This example illustrates the practical process by which expert knowledge and process monitoring information are translated into the structured goal–event representation used in the DML framework, thereby clarifying how the diagnostic reasoning space is constructed in real industrial environments.

#### 3.1.1. Sensor–Function Structural Representation

Sensor observations at time t form the lowest layer of the diagnostic structure.

Sensor set:

$$S(t)\;=\;\{S_{1}(t),\;S_{2}(t),\;\ldots \;,\;S_{q}(t)\}$$

Functional states are derived from sensor measurements:

$$F(t)\;=\;\{F_{1}(t),\;F_{2}(t),\;\ldots \;,\;F_{r}(t)\}$$

Each functional state is computed from groups of sensor measurements:

$$F_{ij}(t)=\;h(S_{ij1}(t),\;S_{ij2}(t),\;\ldots \;,\;S_{ijk}(t))$$

where i indexes the subsystem or functional component, j indexes the sensor within subsystem i, and k indexes the individual sensor inputs contributing to the functional state. This mapping converts raw sensor signals into interpretable functional states using signal processing, statistical analysis, or temporal fuzzy logic.

#### 3.1.2. Goal and Event Evaluation

Goal satisfaction and event risk are evaluated using fuzzy membership functions. Here, the indices m and n represent the number of functional state inputs used to evaluate the goal satisfaction and event risk, respectively.

Goal satisfaction:

$$\mu_{G}(G_{i},t)\;=\;f(F_{i1}(t),\;F_{i2}(t),\;\ldots \;,\;F_{im}(t))$$

Event risk:

$$\mu_{E}(E_{j},t)\;=\;g(F_{j1}(t),\;F_{j2}(t),\;\ldots \;,\;F_{jn}(t))$$

The overall diagnostic state at time *t* is defined as:

$$D(t)\;=\;\{\;\mu_{G}(G_{1},t),\;\ldots \;,\;\mu_{G}(G_{n},t),\;\mu_{E}(E_{1},t),\;\ldots \;,\;\mu_{E}(E_{k},t)\;\}$$

#### 3.1.3. Bayesian Dynamic Truth Relationship

The credibility of causal relationships evolves over time according to observational evidence. Temporal consistency of the diagnostic state can be expressed as:

Pr(D(t) | D(t−1), D(t−2), …)

When repeated observations contradict an assumed relationship, its credibility decreases. Consistent observations increase its probability. Bayesian updating is used to incorporate new evidence:

$$P\left(D\left(t\right)\;|\;E_{t}\right)=\;\frac{P\left(E_{t}\;\right|\;D\left(t\right))*P(D\left(t-1\right))}{P(E_{t})}$$

where $E_{t}$ denotes the evidence derived from sensor observations and functional state evaluations.

#### 3.1.4. LLM-Driven Diagnostic Interaction

When structured rules cannot fully interpret available information, the system invokes LLM modules.

Prompt generation:

$$P\;=\;\pi (D\left(t\right))$$

LLM inference result:

R = LLM(P, T)

T represents contextual knowledge sources such as maintenance records, engineering documentation, domain literature, and operational databases.

#### 3.1.5. Deterministic Normalization and Stability Control

LLM outputs are not used directly for decision-making. Instead, they are transformed into normalized inference signals using deterministic functions.

Let R denote the raw LLM response and C the extracted confidence score. The normalized signal is:

$$R_{n}\;=\;N(R,\;C)$$

where N(·) is a deterministic normalization function that converts semantic outputs into bounded fuzzy updates.

To ensure reasoning stability, the following constraint is enforced:

| D(t)−D(t−1) | < τ

where τ is a stability threshold limiting the influence of external inference signals.

#### 3.1.6. Unified Hybrid Diagnostic Equation

The hybrid diagnostic reasoning process can be summarized as:

$$D\left(t\right)=f\left(D\left(t-1\right),\;S\left(t\right),\;F\left(t\right),\;R_{n}\right)$$

where

- $S\left(t\right)$: sensor observations at time *t*
- F(t): functional states derived from sensors at time t
- $R_{n}$: normalized LLM inference signal
- $D\left(t-1\right)$: previous diagnostic state

This formulation integrates symbolic causal reasoning (DML), probabilistic updating (Bayesian inference), and semantic reasoning (LLM interaction).

#### 3.1.7. Formal Diagnostic Model

To formally describe the reasoning mechanism, the diagnostic state is modeled as a dynamic probabilistic structure integrating sensor observations, functional states, and causal relationships.

Let

$S_{ij}(t):$ sensor state j in subsystem i at time t
$F_{ij}(t)$: functional state derived from sensors
$D_{ij}(t)$: overall system diagnostic state

Functional states are derived from sensor measurements through deterministic mappings:

$$F_{ij}\;(t)\;=\;\Phi_{ij}(S_{ij}\left(t\right))$$

The diagnostic state evolves according to probabilistic belief propagation:

D(t) = P(S(t) | S(t−1), S(t−2), …)

Bayesian belief updating adjusts the credibility of causal relationships:

$$P_{new}\left(R_{k}\right)\propto \;P\left(E_{t}\;\right|\;R_{k})\;P_{prior}(R_{k})\;$$

where $R_{k}$ denotes a candidate’s causal relationship and $E_{t}$ represents new observational evidence.

Failure conditions are represented using fuzzy membership functions:

$$\mu_{i}\;=\;\mu_{i}(S_{ij}(t))$$

The normalized LLM signal is incorporated into the state update:

$$D(t)\;=\;f(D(t-1),\;S(t),\;F(t),\;R_{n})$$

A stability constraint ensures bounded updates:

$$|\;D\left(t\right)\;-\;D\left(t-1\right)\;|\;<\;\tau$$

This formulation summarizes the hybrid reasoning process in which the diagnostic state evolves through deterministic DML inference, Bayesian belief updating, and normalized LLM semantic interpretation. The hybrid diagnostic workflow is illustrated in Figure 3c, where sensor observations are mapped to functional states that update the diagnostic state $D_{t}$ through Bayesian inference and LLM interpretation.

## 4. Logic-Driven LLM Orchestration via a DML Backbone

### 4.1. Platform Architecture and Execution Flow

**Figure 4:**
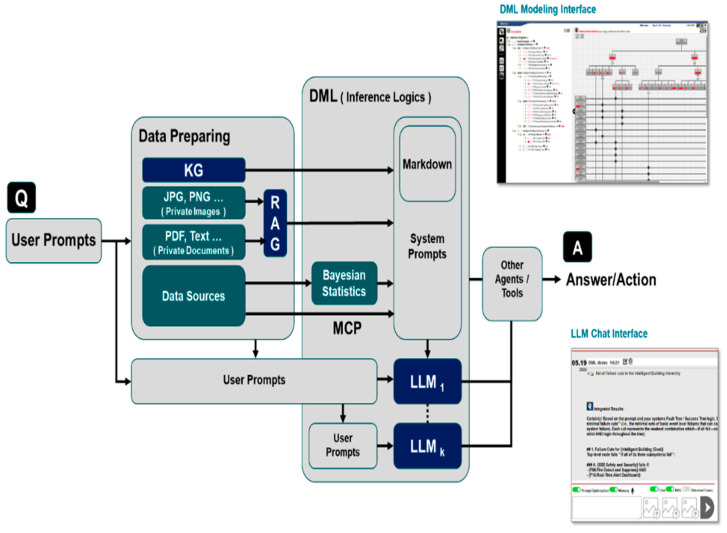
The DML–LLM platform orchestrates data preparation, RAG/KG retrieval, Bayesian statistics, and DML inference, routing system prompts to specialized LLMs/tools and logging fused evidence for auditable answers and human-in-the-loop review.

The hybrid framework centers on the DML engine, which orchestrates data retrieval, preprocessing, and belief updates by activating relevant goal/event nodes. As evidence is gathered, DML checks whether all necessary inputs for the active nodes are available. If information is missing or incomplete, DML sends a targeted request to another component, such as a retrieval tool or an internal LLM, to supply only the specific interpretation needed. In this way, LLM involvement remains selective and is triggered only when the execution pipeline encounters an information gap. Bayesian estimators are treated as modular belief-update components within the DML execution flow, rather than standalone probabilistic decision engines. Figure 4 illustrates the DML–LLM platform architecture.

In practice, LLM modules are invoked only when DML reasoning encounters unresolved semantic information. In most diagnostic cycles, the inference is completed entirely within the deterministic DML engine, which significantly reduces runtime overhead and ensures practical deployment feasibility.

### 4.2. Causal–Temporal Reasoning Within the DML Engine

**Figure 5:**
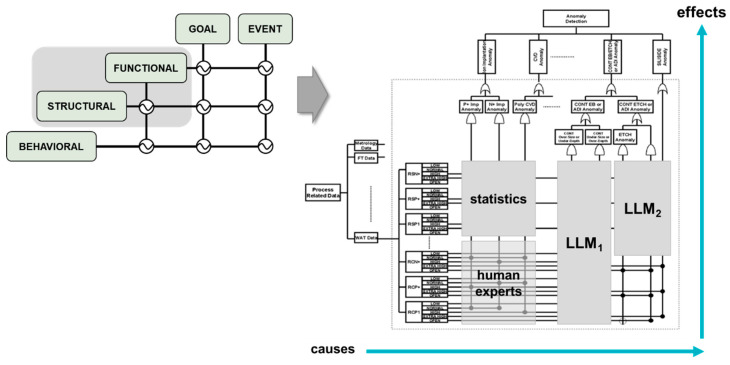
DML goal and event lattice supporting evidence routing, integration with statistical and expert tools, and targeted use of LLM modules.

DML organizes system knowledge into connected nodes that represent goals, functions, physical structures, and system behaviors across time. These nodes define how evidence moves through the model and how different components influence one another. As illustrated in Figure 5, the goal and event lattice provides a routing structure that links functional, structural, and behavioral logic, allowing DML to integrate statistical tools, human expertise, and specialized LLM modules within a unified reasoning pipeline. Using this structure, DML can reason forward from a potential cause to predict downstream effects or reason backward from an observed symptom to identify likely contributors.

### 4.3. Internal LLM Role: Logic-Guided Inference and Knowledge Completion

LLMs play an internal and selectively invoked role, activated only when the DML engine identifies unresolved uncertainty, the need to interpret unstructured or weakly structured data, or a requirement for higher-level semantic inference. Based on current measurements and evolving node states, the DML engine determines whether an LLM should be invoked. When needed, it selects task-specific prompts, contextual constraints, relevant RAG databases, structured inputs, and external APIs.

In such cases, DML constructs a prompt that encapsulates the current system state and procedural instructions, and packages it in a fixed and explicit markdown format so that the LLM receives only the information necessary for the targeted reasoning task. The prompt explicitly specifies the role of the active DML node, the missing or ambiguous information to be resolved, and the logical or procedural rules that must be followed. LLM outputs are not treated as final decisions. Instead, they are converted into quantitative or fuzzy state updates for downstream DML nodes. These updated states may, in turn, serve as inputs to other nodes, enabling dynamic, iterative, and temporally consistent reasoning across the DML graph. By constraining LLM interactions using predefined templates and logic-driven orchestration, the framework limits speculative reasoning.

**Figure 6:**
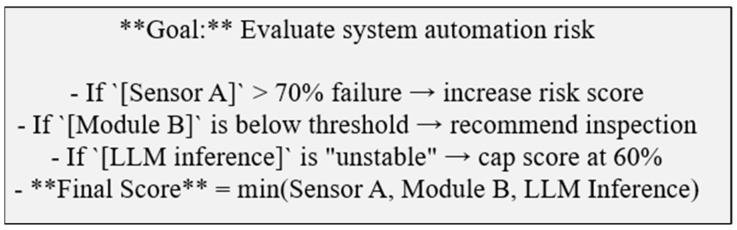
Simplified DML template showing how LLM inputs are constrained and normalized before entering the reasoning process.

The output produced by the LLM is expressed in a controlled form, such as a classification label, a confidence score, or a brief diagnostic explanation. This output is then passed to a deterministic function node within the DML framework. The function node applies operations such as weighting, stability checks, thresholding, or fuzzy logic to convert the LLM’s semantic inference into a representation suitable for causal and temporal reasoning. When the LLM output is unstable or internally inconsistent, its influence can be limited or ignored entirely. In this way, LLM-based inference serves to augment, rather than override, the deterministic and explainable structure of the DML. Figure 6 highlights the role of DML templates in constraining and validating LLM outputs prior to causal–temporal reasoning.

### 4.4. External LLM Role: Human Explanation and Interaction Layer

After the DML framework completes its diagnostic reasoning and reaches a conclusion, an external LLM can be used to translate the results into natural language. This external LLM does not participate in the diagnostic decision-making process and does not introduce new information into the reasoning chain. Instead, it functions as a communication layer that presents explanations, clarifies evidence pathways, and retrieves relevant supporting documentation when required. Since the DML engine records each evidence source, rule activation, and belief update, the external LLM can leverage this provenance information to generate a clear and structured explanation of the diagnostic process in natural language. This allows operators and analysts to understand which signals contributed to the conclusion and the rationale behind it.

This clear separation between internal inference and external explanation preserves both transparency and interpretability. Operators can examine how and why a conclusion was reached without affecting the deterministic behavior of the underlying logic. All decisions and evidence contributions are logged in real-time, creating an auditable record suitable for expert review and regulatory compliance. Importantly, LLM-generated outputs never directly initiate alarms or control actions, as all actuation decisions remain governed by DML rules and traceable evidence.

In summary, the internal LLM operates *within* the diagnostic reasoning loop under deterministic DML constraints and can influence node belief states only through validated transformations. In contrast, the external LLM operates strictly *after* reasoning is complete, serving only as a communication layer without modifying system state or inference pathways.

Table 2 summarizes this contrast. Pure LLM pipelines excel at handling unstructured information and delivering rapid insights, but they lack consistent inference paths and do not support the level of governance needed in high-reliability manufacturing settings. The hybrid approach preserves these advantages while adding traceable logic layers, stable decision rules, and structured intermediate steps.

## 5. Case Study: FAB Process Integration FDD

This case study presents a typical diagnostic workflow used by FAB Process Integration Engineers (PIEs) and illustrates how a DML–LLM hybrid architecture enhances fault detection, diagnosis, and prediction in advanced semiconductor manufacturing, where process complexity and data volume continue to increase. In mature technology nodes (e.g., ≥40 nm), a standard 25-wafer lot typically involved 200–500 process steps. Under these conditions, sampling-based statistical methods and structured parameter monitoring were generally sufficient for yield surveillance and root cause analysis (RCA) of low-yield excursions or WAT failures. Process deviations could often be identified by correlating a limited set of inline measurements, SPC charts, and electrical test results.

As technology nodes advanced below 28 nm—and particularly at ≤16 nm—the scope and complexity of PIE diagnostics expanded significantly. A single lot may now traverse 1000–2000 tightly coupled process steps, and when wafer maps, inline metrology, equipment logs, recipe histories, and defect inspection images are fully retained, the data volume per lot can reach hundreds of gigabytes (e.g., 300–800 GB). Historically, only sampled or aggregated data were archived, limiting the resolution and traceability of RCA for yield loss events.

**Figure 7:**
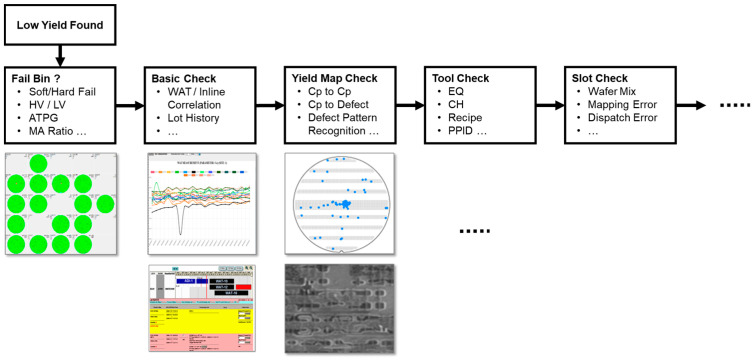
Multimodal workflow for low-yield diagnosis by Process Integration Engineers (PIEs).

In modern fabs, PIEs must routinely interpret multimodal and heterogeneous evidence, as illustrated in Figure 7, including wafer-level defect maps, slot-based inline trends, WAT signatures, equipment event logs, and high-resolution SEM images. These data sources are semi-structured or unstructured, making manual correlation across process steps, tools, and wafers increasingly impractical under real-time production constraints.

To address this challenge, LLM-based RAG pipelines are used to filter, retrieve, and summarize the most relevant diagnostic evidence. Rather than replacing engineering judgment, these tools support PIEs by highlighting anomalous patterns, cross-tool correlations, and historically similar cases, allowing experts to focus on the most plausible fault mechanisms.

At the same time, semiconductor manufacturing remains governed by well-established physical principles, equipment behaviors, and process integration heuristics accumulated over decades of fab operation. In the proposed framework, these principles are encoded within DML structures, which determine when and how LLMs are invoked—specifying which prompts to issue, which data sources to retrieve, and which fault hypotheses to prioritize under specific operating conditions. DML thus provides a causal and procedural backbone that constrains and guides language-based reasoning.

In production deployment, this hybrid DML–LLM workflow demonstrated substantial improvements in diagnostic efficiency. Time to root cause was reduced and case-closure rates increased from 54.4% to 75%, resulting in measurable gains in yield recovery and throughput. LLM-assisted summarization further reduced the reporting burden on PIEs and improved on-time delivery from 89.8% to 99.3%. To structure Process Integration-oriented FDD, we adopt a taxonomy linking key diagnostic tasks to system-level objectives and supporting capabilities. The complete taxonomy is provided in Appendix A (Table A1).

### 5.1. Stage 1 Pre-Training: Data-Driven, State-Aware Orchestration from Correlation Screening to Causal Rule Initialization

Due to variations in product type, technology node, device architecture, and process integration schemes, semiconductor manufacturing recipes differ significantly across fabs and products. Consequently, FDD systems cannot rely on fixed or globally tuned diagnostic logic. State-aware pre-training is therefore required to establish a reliable baseline for automated diagnosis.

In Stage 1, diagnostic logic is initialized using data-driven statistical evidence derived from production-stable reference lots. In practice, the first 20 qualified “good lots” for a given product–tool–recipe configuration are selected as the baseline population and used to calibrate diagnostic logic weights before anomaly-triggered reasoning is activated. The first 20 confirmed “good lots” are used because they represent a stable production period verified by process engineers and RCA reports, and this range is consistent with common SPC/FDC baseline calibration windows of approximately 10–30 stable lots.

**Figure 8:**
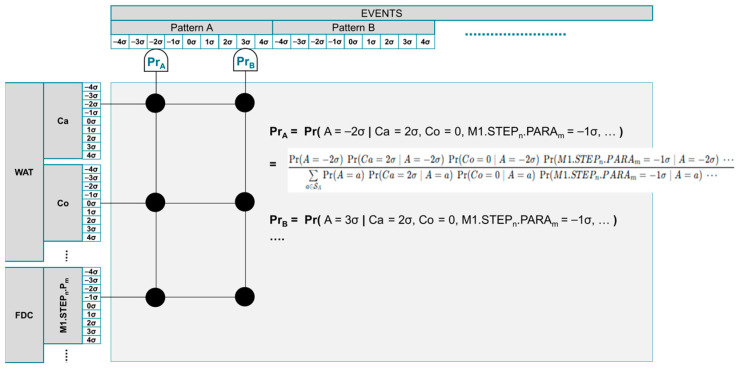
Bayesian updating estimates the conditional likelihood of observed WAT/inline deviations given a target failure mode under specific tool–recipe contexts, producing adaptive priors for downstream diagnosis.

For each target diagnostic objective, such as a specific low-yield mode or defect pattern, the system evaluates historical occurrences under constrained conditions, including specific tools, chambers, process steps, and production parameters. A Bayesian updating scheme (Figure 8) estimates the conditional likelihood of observing these deviations given the target failure mode under specific contexts. These likelihoods are translated into adaptive logic weights that serve as priors for downstream diagnosis.

As shown in Figure 8, the interface displays the target WAT parameter with its associated fuzzy sets, representing soft operational states rather than hard thresholds. Correlated WAT parameters and in-process metrology signals are listed alongside wafer- and slot-level measurement distributions, enabling fine-grained analysis within each 25-wafer lot. As illustrated in Figure 8, ${Pr}_{A}$, ${Pr}_{B}$, … denote conditional likelihoods under given constraints, which are interpreted as the degree of truth of the corresponding relationships in fuzzy logic.

Correlations that are validated through this process are formalized as initial causal rules within the DML framework, with probabilistic weights assigned to reflect historical evidence, uncertainty, and contextual relevance. This step represents a shift from exploratory correlation screening to causally informed diagnostic reasoning. By anchoring LLM-assisted inference in statistically calibrated and expert-validated logic, Stage 1 pre-training ensures that subsequent diagnostic reasoning remains traceable, interpretable, and consistent with underlying physical process behavior, rather than being influenced by spurious correlations or transient data patterns.

### 5.2. Stage 2: DML-Based Rule Generation and Validation

Once a low-yield event is detected, the next challenge is to determine why it occurred. The DML–LLM diagnostic workflow begins by identifying which process or metrology parameters show the strongest statistical association with the observed yield shift. These parameters are ranked by importance and then grouped through correlation clustering to reveal signals that tend to drift together across wafers or lots. The ranked parameters and clusters are then mapped into a DML diagnostic graph, where the engine checks whether these statistical patterns align with known functional dependencies and temporal ordering. A typical example is when RAMP_DOWN2 and INPUT_VOLTAGE exhibit a stable co-drift pattern across multiple wafers together with a characteristic defect signature, leading to a candidate rule such as:

**IF** (RAMP_DOWN2 is **Low** with membership μ_1_)
**AND** (ZONE_2_VOLTAGE is **High** with membership μ_2_)
**THEN** the risk of yield loss increases with degree μ = f (μ_1_, μ_2_).

Unlike a simple SPC heuristic, this rule is evaluated through graded fuzzy memberships rather than crisp thresholds. The resulting rule activation represents a degree of truth, quantifying how strongly the observed temporal pattern supports an increased risk of yield loss. This interpretation is consistent with the causal and temporal structure defined in the DML graph, enabling soft evidence accumulation instead of binary decision-making.

**Figure 9:**
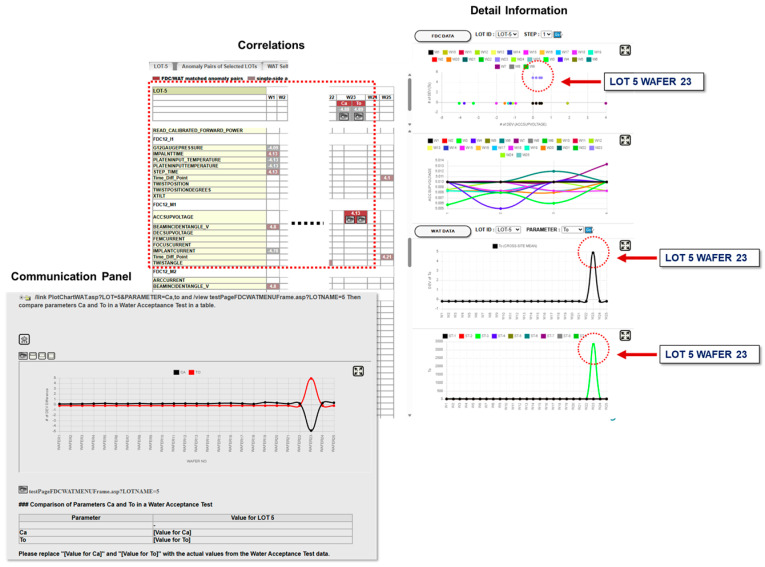
Example of a DML–LLM conversational console showing Lot 5 WAT co-drift at To. The system uses real-time dialog to rank risk paths, link them to SOPs and Golden Rules, generate targeted action plans, and log the full decision chain for future audit and reuse.

Engineers review these candidate rules through the visual interface shown in Figure 9, where parameter hierarchies (left), correlation matrices and clusters (center), and drift visualizations such as scatter plots linking RAMP_DOWN2 and INPUT_VOLTAGE or trend charts and box plots showing excursion behavior (right). These diagnostic rules are then subjected to human-in-the-loop validation. Only those approved by PIEs are promoted to “Golden Rules” in the DML knowledge base. This integration of statistical discovery, causal–temporal validation, and human verification produces diagnostic rules that are both interpretable and operationally reliable.

Figure 8 and Figure 9 together describe the construction and calibration of the DML model, where Figure 9 establishes the diagnostic rule structure and Figure 8 calibrates rule confidence using Bayesian updating, both occurring prior to runtime inference and without LLM involvement.

### 5.3. Stage 3: AI Prediction: Proactive Risk Assessment and Action Planning

Prediction extends diagnosis by identifying early drift patterns that precede excursions. In the conversational console shown in Figure 9, the system highlights co-drift signals, retrieves similar past incidents, and uses temporal fuzzy reasoning to rank likely process or equipment paths.

In the Lot 5 WAT example, the console reveals a co-drift pattern in which the WAT capacitance-related parameter $C_{a}$ trends downward while the oxide-thickness–related parameter $T_{o}$ trends upward, with Wafer 23 exhibiting the strongest deviation. Although these deviations remain below traditional SPC alarm limits, the DML prediction engine identifies them as high-risk precursors and presents supporting evidence. This evidence includes time-series trend charts, lot-to-tool linkage matrices, and historical analogs retrieved through RAG. Using this collected information, the system generates a ranked list of candidate risk paths. It maps the early drift patterns to possible mechanisms such as upstream thermal control drift, chemical concentration imbalance, or maintenance-related degradation. The retrieval layer provides previously resolved incidents, and the LLM, operating within DML constraints, produces an action plan within the conversational interface. This plan includes likely causes with ranked weights, verification steps, expected risk windows, and links to Golden Rule identifiers and relevant operating procedures.

Engineers can act directly on these recommendations without manually cross-checking SPC charts or maintenance logs. In this example, the team performed targeted checks on the gas supply temperature loop within six hours, which prevented an estimated yield loss on the order of ten percent based on historical excursion impact analysis before a full excursion developed.

All dialog, retrieval steps, measurements, and resulting actions are automatically logged. This creates a complete and auditable decision trail that is incorporated back into the DML knowledge base, which strengthens future early warning accuracy and response efficiency.

### 5.4. Quantitative Evaluation and Results

This section evaluates the performance of the proposed DML–LLM hybrid architecture in semiconductor FDD, comparing it against two baselines: traditional SPC/FDC rule-based workflows and a pure LLM pipeline with retrieval-augmented reasoning. The evaluation focuses on three research questions: whether the hybrid system shortens time to anomaly detection and root cause localization; whether it improves diagnostic precision and recalls while maintaining interpretability; and whether it strengthens traceability and auditability in real fab settings.

The study uses a confidential six-month dataset from a Process Integration Engineering (PIE) group in a mature logic fab (≈40 nm). While the evaluation is conducted on a mature node for confidentiality and controlled benchmarking, the proposed workflow is designed to scale to advanced-node settings where multimodal data volume and process coupling are substantially higher.

The evaluation dataset consists of 117 production lots collected over six months from a mature logic semiconductor FAB. Each lot contains approximately 1300 process steps, 45 inline metrology parameters, and more than 3200 SPC/FDC monitoring channels. Ground truth labels were obtained from documented yield excursion investigations performed by Process Integration Engineers (PIEs). Each diagnostic case includes confirmed root cause reports validated through standard RCA procedures. Three diagnostic configurations were evaluated under identical conditions:

1. Conventional SPC/FDC workflow with manual triage
2. Pure LLM + RAG reasoning pipeline
3. Proposed DML–LLM hybrid architecture

All methods were applied to the same dataset to ensure consistent benchmarking conditions.

Performance was measured using metrics, including Time to Detect (TTD), Time to Root Cause (TTRC), diagnostic precision, recall, F1 score, provenance completeness, and engineer triage time. The responsiveness results, presented in Table 3, show that the hybrid system detects yield-impacting anomalies substantially earlier than the baselines. It reduces TTD from 7.4 h in SPC/FDC and 3.1 h in the pure LLM pipeline to 1.2 h. Root cause localization follows a similar trend, with TTRC decreasing from 15.2 h and 8.9 h to 4.7 h. These gains come primarily from the temporal fuzzy logic gates, which surface subtle drift patterns that traditional SPC thresholds or free-form LLM reasoning fail to capture. The evaluation metrics are defined as follows:

Time to Detection:

$$TTD=t_{detect}-t_{fault}$$

Time to Root Cause:

$$TTRC=t_{rca}-t_{fault}$$

Provenance Completeness:

$$PC=\frac{N_{traceable}}{N_{total}}$$

Diagnostic accuracy metrics are summarized in Table 4. The hybrid system achieves higher precision, recall, and F1 scores than both baselines, demonstrating that tighter integration of causal reasoning with statistical and semantic cues leads to more reliable conclusions. The hybrid approach preserves interpretability because every LLM contribution is constrained by explicit DML pathways, preventing unsupported or speculative inferences.

Governance and effort-related metrics appear in Table 5. The hybrid system provides near-complete provenance, recording 96 percent of decision steps compared to 18 percent for SPC/FDC and 42 percent for the pure LLM system. It also reduces the time engineers spend on triage from 72 min per event in the baseline to 18 min. These improvements arise from the system’s structured evidence logging and from DML’s ability to narrow the analysis space before LLMs are invoked.

### 5.5. End-to-End Diagnostic Walkthrough

To complement the quantitative evaluation presented in the previous subsections, this final subsection provides an end-to-end diagnostic walkthrough of a representative lot-level case. While the preceding results demonstrate improvements in responsiveness, diagnostic accuracy, and governance, the following example focuses on execution: how the DML–LLM hybrid architecture operates step by step in a concrete diagnostic instance. The example illustrates how structured sensor evidence, temporal fuzzy reasoning, targeted LLM invocation, and belief updating interact within a single diagnostic cycle.

The baseline comparisons in Table 3, Table 4 and Table 5 also clarify the contribution of individual modules. The improvement from SPC/FDC to the pure LLM pipeline reflects the gain from language-based semantic interpretation of unstructured logs and historical cases. The further improvement from the pure LLM pipeline to the full DML–LLM hybrid system isolates the contribution of the DML backbone, including fuzzy soft-threshold reasoning and temporal persistence operators. Fuzzy logic improves sensitivity to gradual near-threshold drift, while temporal reasoning enhances stability by suppressing transient fluctuations. Together, these structured reasoning mechanisms account for the earlier detection and higher precision observed in the hybrid system.

#### 5.5.1. Initial Observation and Event Triggering

Consider a production lot (Lot 5) processed under a stable product–tool–recipe configuration. During routine monitoring, no hard SPC or FDC limits are violated. However, temporal analysis of wafer-level data reveals a gradual co-drift pattern: the WAT parameter *Ca* exhibits a sustained downward trend, while *To* shows a compensatory upward drift over several consecutive wafers. Although each parameter remains within nominal control limits, the joint pattern persists beyond a predefined temporal window.

These observations are ingested by the DML engine and transformed into fuzzy sets (e.g., *Ca* is Decreasing, *To* is Increasing) with graded membership values. The corresponding structural and behavioral nodes in the DML graph are partially activated based on these fuzzy memberships and temporal persistence criteria.

#### 5.5.2. DML Node Activation and Causal Routing

The activated behavioral nodes propagate evidence upward through the DML hierarchy toward relevant functional and event nodes associated with thermal regulation and process stability. Temporal fuzzy operators evaluate whether the observed co-drift is transient or persistent. Because the deviation is sustained across wafers and aligns with known functional dependencies, the DML engine raises the activation level of a candidate *early yield risk* event node.

At this stage, DML determines that numerical and rule-based evidence alone is insufficient to discriminate among several plausible causal paths (e.g., upstream thermal control drift, gas delivery imbalance, or maintenance-related degradation). This unresolved uncertainty triggers a controlled request for semantic interpretation.

#### 5.5.3. Logic-Guided LLM Invocation

Based on the current DML state, the engine selects an internal LLM module specialized for contextual interpretation of maintenance logs and historical incident summaries. DML constructs a task-specific prompt using a fixed markdown template that includes: the identity and role of the active DML node, the observed fuzzy–temporal pattern requiring clarification, the specific information gap to be resolved, and explicit constraints prohibiting the creation of new causal relationships.

Relevant historical cases and maintenance records are retrieved via an RAG pipeline and injected into the prompt as a bounded context. No raw sensor streams or unrelated documents are exposed to the LLM.

#### 5.5.4. LLM Output Normalization and Fuzzy State Update

The LLM returns a constrained inference signal, indicating that similar co-drift patterns in prior cases were frequently associated with gradual degradation in a gas supply temperature control loop following recent maintenance activities. The output includes a short semantic rationale and a confidence estimate.

This result is not treated as a diagnosis. Instead, it is passed into a deterministic DML function node, where the confidence score is translated into a fuzzy belief update for the corresponding structural and behavioral nodes. Stability checks and weighting rules ensure that the update is consistent with existing causal constraints. If the output were inconsistent or unstable, its influence would be reduced or discarded.

#### 5.5.5. Iterative Reasoning and Diagnosis Formation

With the updated fuzzy states, DML propagates revised beliefs through the causal–temporal graph. Competing hypotheses are reweighted, and the most plausible diagnostic path emerges with an associated confidence level. In this example, the system identifies an elevated risk of yield loss linked to upstream thermal control drift, several hours before a conventional SPC alarm would have triggered.

The final diagnostic conclusion includes the ranked fault hypothesis, supporting evidence paths, temporal context, and associated confidence values. Intermediate steps such as sensor evidence, fuzzy memberships, LLM invocation, belief updates, and rule activations are logged to form a complete and auditable reasoning trail.

#### 5.5.6. Explanation and Human Interaction

Once the diagnosis stabilizes, an external LLM is optionally invoked to translate the DML reasoning trace into a human-readable explanation for PIEs. This external model does not alter the diagnosis; it merely summarizes why the conclusion was reached, which signals were critical, and which verification actions are recommended.

Through this end-to-end example, the execution flow of the DML–LLM hybrid architecture becomes explicit: DML governs state evaluation, temporal reasoning, and causal routing, while LLMs are selectively invoked to resolve semantic uncertainty and return controlled inference signals. The result is a dynamic yet traceable diagnostic process that combines early detection capability with audit-ready explainability.

## 6. Discussion and Outlook

The quantitative evaluation demonstrates that the DML–LLM hybrid architecture does more than improve diagnostic metrics. It changes how FDD operates in practice. The system identifies anomalies several hours earlier, narrows root causes with higher precision, and provides near-complete provenance trails that are absent in SPC/FDC or pure LLM workflows. These improvements reduce engineering workload, shorten response cycles, and strengthen confidence in automated diagnostic support.

**Figure 10:**
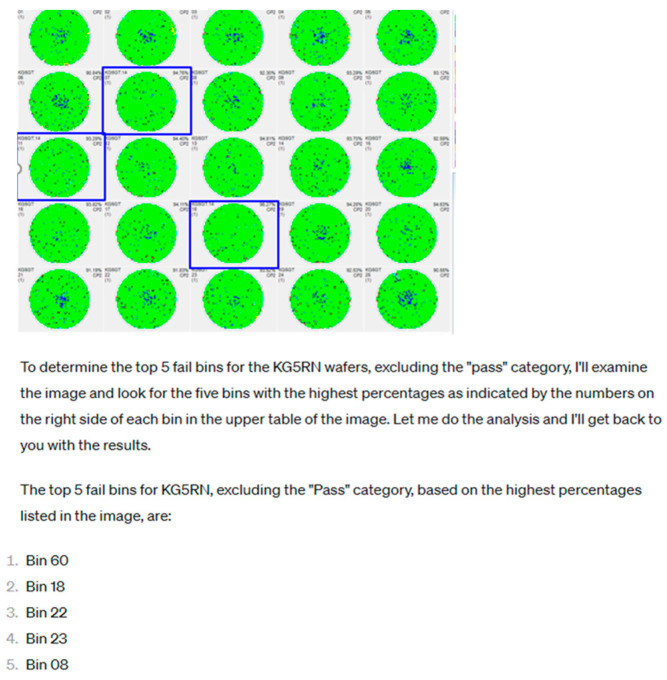
Pure LLM pipeline provides quick interpretation of unstructured data with minimal engineering effort, but it lacks explicit traceability and causal structure.

Large language models have reshaped how engineering knowledge is accessed and communicated. Pure LLM pipelines can interpret unstructured inputs such as wafer maps, text logs, or alarm messages with minimal engineering effort, enabling rapid prototyping of diagnostic tools. Figure 10 illustrates this capability: a wafer map anomaly detector that previously required extensive coding can now be replicated through prompt-based instructions, producing immediate visual interpretation and natural language summaries. These strengths, including fast deployment, flexible data handling, and expressive explanations, make pure LLM approaches appealing for early exploratory analysis.

**Figure 11:**
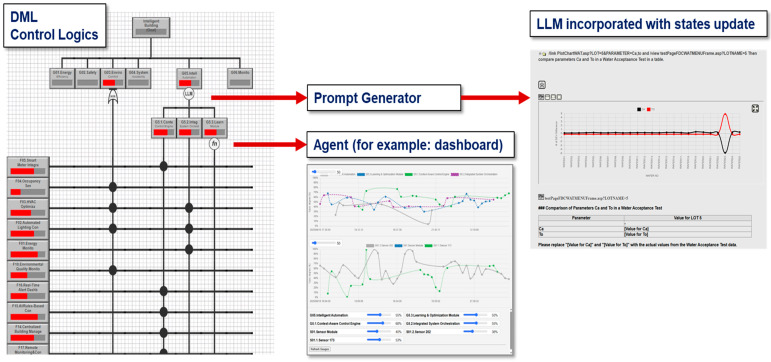
DML–LLM hybrid pipeline incorporates domain rules and temporal logic, enabling structured reasoning, explainable diagnostics, and governance-ready traceability.

However, engineering workflows also require stable reasoning, explicit causal structure, and full traceability. Pure LLM systems do not inherently encode process logic or temporal dependencies, and their output may vary depending on prompt design or input phrasing. Figure 11 shows how the DML–LLM hybrid architecture addresses these challenges. DML provides a structured causal–temporal backbone that governs how evidence propagates, which dependencies matter, and how decisions are reached. LLM modules operate within this backbone to interpret unstructured inputs and generate explanations, but the logical steps remain explicit and auditable. The result is a reasoning system that retains the flexibility of language models while grounding each inference in a transparent and verifiable structure.

### 6.1. Limitations

The DML–LLM hybrid architecture improves diagnostic accuracy, traceability, and interpretability, but several limitations must be addressed before it can be deployed at scale. A primary challenge is the construction and maintenance of DML models. These models must encode detailed causal and temporal relationships, which often require extensive input from experienced engineers. While parts of this process can be automated through feature extraction or pattern-mining methods, expert supervision remains essential, particularly in safety- and yield-critical manufacturing environments where incorrect logic can misrepresent failure mechanisms.

A second limitation concerns the system’s ability to operate at scale. Modern fabs generate vast amounts of structured, semi-structured, and unstructured data, and the retrieval pipelines that support multimodal analysis can become bottlenecks. Efficient indexing, memory management, and streaming interfaces are required to maintain real-time responsiveness. Similar constraints arise on the LLM side. Large prompts, long context windows, and multimodal reasoning introduce non-trivial latency and computational cost, which can restrict deployment in high-throughput settings where takt times are tight.

The quality of unstructured evidence also presents risks. Technician notes, maintenance logs, and AOI annotations may be incomplete or inconsistent, limiting the reliability of LLM-generated interpretations. Prompting and verification layers help to mitigate this but cannot fully compensate for missing or noisy human-generated inputs. Moreover, LLM outputs may vary based on prompt design or linguistic ambiguity, making consistency a continual concern and necessitating DML-based validation layers.

Another important limitation concerns modeling bias and parameter sensitivity. Because DML structures are partially expert-defined, there is a risk that embedded assumptions may reflect subjective bias or incomplete domain understanding. Similarly, the specification of fuzzy membership functions and Bayesian priors can influence belief propagation behavior, particularly in edge cases or under limited data conditions. While validation and iterative calibration can mitigate these risks, formal sensitivity analysis is not included in the present study. In addition, reliance on external LLM providers introduces practical considerations related to model updates, API stability, and long-term availability. Although architecture constrains LLM outputs within deterministic validation layers, deployment in safety-critical environments may require controlled model versioning or locally hosted alternatives to ensure reproducibility and governance.

Another limitation is the system’s sensitivity to process drift. Tool aging, recipe retargeting, chamber seasoning, and the emergence of new defect modes can gradually erode the validity of previously learned DML structures or rule thresholds. Ensuring long-term reliability requires periodic retraining, rule revision, and automated drift monitoring to identify outdated knowledge structures.

Integration with legacy infrastructure adds further complexity. For example, many fabs rely on Manufacturing Execution System (MES), SPC, and FDC systems that predate modern AI architectures and expose limited interoperability. Deploying the hybrid framework in production requires adapters, ontology mapping tools, and schema translators. These components add engineering effort and can create operational fragility if they are not carefully managed.

Finally, although the hybrid system is designed for human-in-the-loop operation, this reliance on expert review introduces its own constraints. Engineers remain responsible for validating DML structures, reviewing candidate diagnoses, and approving recommended actions. While this oversight maintains trust and interpretability, it also increases cognitive load and may limit scalability unless supported by effective alert prioritization and interface design. Overall, these limitations highlight the need for continued research in automated logic extraction, efficient multimodal retrieval, LLM consistency mechanisms, and robust integration pathways. Addressing them is essential for achieving reliable, scalable deployment of hybrid reasoning architectures in production semiconductor environments.

### 6.2. Future Work

Fully realizing the capabilities of the DML–LLM hybrid architecture will require advances on several fronts, ranging from model construction to system-level integration. A particularly promising direction is the use of semi-automated workflows for building DML models. Instead of manually defining the entire causal structure, historical logs, recurring correlation patterns, and previous incident reports could be used to propose candidate causal and temporal relationships. Domain experts would then focus on validating and refining these candidates. This shift would substantially reduce the effort required to develop and maintain DML models, especially in large and evolving manufacturing environments.

The performance of multimodal data retrieval also remains a practical concern. Modern fabrication facilities generate data streams that are not only large in volume but also diverse in structure and timing. Maintaining responsive retrieval will require hierarchical indexing, compact embedding representations, and real-time data interfaces. Without such improvements, early warning detection becomes difficult, particularly in high-mix, low-volume settings where process states change frequently.

Reasoning reliability presents a different set of challenges. As the system grows in scope, maintaining alignment between inferred conclusions and established domain knowledge becomes increasingly important. Techniques such as constrained prompting, adaptive prompt adjustment, and automated consistency checks may help keep reasoning bounded by known causal relationships and reduce the risk of unsupported inferences. Long-term process drift adds another layer of complexity. Equipment aging, recipe updates, and operational changes can gradually invalidate previously learned causal rules. Detecting these shifts automatically would allow the DML model to be updated before diagnostic accuracy degrades.

Deployment considerations extend beyond reasoning and retrieval. Many fabrication facilities operate on legacy MES, SPC, and FDC platforms that were not designed with hybrid reasoning architectures in mind. Integrating the proposed framework into these environments will require middleware that can accommodate heterogeneous data formats and vendor-specific interfaces while preserving data fidelity and operational stability.

Although this work is grounded in semiconductor manufacturing, the underlying ideas are not domain-specific. Similar diagnostic challenges arise in other safety-critical and high-reliability fields, including aerospace, energy systems, and healthcare. Applying the architecture in these contexts would provide insight into its generality and help identify which components require adaptation. Such studies could also inform broader design principles for hybrid diagnostic systems that combine structured causal reasoning with learned representations.

An important direction for future work is an evaluation of the hybrid architecture under adversarial and stress-testing scenarios. This includes analyzing the behavior of unconstrained LLM outputs versus DML-constrained outputs, measuring hallucination rates under prompt perturbations, and evaluating failure containment mechanisms in safety-critical workflows.

A promising additional direction is the use of Small Language Models (SLMs). Large Language Models are powerful but require significant computational resources [38], which makes them difficult to deploy in real industrial environments. Small Language Models are becoming more capable as they are trained for specific domains with less computational resources [39,40]. Since these smaller models can run on local devices and operate in real-time, they could be integrated directly into digital twins and DML structures. In practical deployments, however, SLMs would require task-specific fine-tuning and careful evaluation to verify that their output remains consistent with process logic and domain constraints. With appropriate adaptation, SLMs could interpret operator logs, explain anomalies using local context, and perform basic causal reasoning that follows established DML relationships. Incorporating these smaller models has the potential to make the diagnostic system more scalable, transparent, and feasible to deploy in safety-critical environments.

Finally, future development should focus on assurance and certification. Standardized testing procedures, safety cases, and auditing frameworks will be essential for regulatory acceptance. These steps will help move the DML–LLM system from a research prototype to a certifiable diagnostic platform suitable for mission-critical environments.

## 7. Conclusions

This work presented a hybrid diagnostic architecture that integrates DML with LLMs to address the growing complexity of FDD in semiconductor manufacturing. By combining explicit causal–temporal structure with flexible semantic interpretation, the DML–LLM framework bridges the gap between traditional rule-based analytics and modern language model intelligence. The case study demonstrates that this hybrid approach delivers substantial improvements in responsiveness, diagnostic accuracy, and traceability compared with both SPC/FDC systems and pure LLM pipelines. Early anomaly detection is enabled by temporal–fuzzy reasoning, while DML routing constrains LLM outputs to valid causal pathways, resulting in explanations that are both interpretable and auditable.

Beyond performance gains, the results highlight an important shift in how engineering knowledge can be represented and operationalized. Pure LLMs offer speed and flexibility but lack the consistency and governance required for high-reliability environments. DML counterbalances these limitations by providing a stable logical backbone, ensuring that every inference step remains transparent and verifiable. Together, the two components form a complementary reasoning system that supports human engineers as a second opinion advisor rather than replacing expert judgment.

The proposed architecture provides a practical foundation for deploying trustworthy AI in mission-critical manufacturing settings, where decisions must remain defensible and aligned with established engineering practice. As future work advances semi-automated DML construction, faster multimodal retrieval, improved consistency checks for LLMs, and domain-level assurance frameworks, the hybrid approach has the potential to become a certifiable standard for diagnostic reasoning, not only in semiconductor fabrication but across other safety-critical industries. While the framework is evaluated on semiconductor manufacturing data, its underlying design principles, namely the integration of structured causal and temporal reasoning with constrained LLM interpretation, are not domain-specific. With appropriate adaptation of the DML model and supporting data interfaces, the architecture can be extended to other high-reliability domains that rely on distributed sensor networks, such as industrial IoT and large-scale monitoring systems.

Future work will focus on making the framework easier to scale and maintain in real production environments. This includes streamlining the construction and updating of DML models, improving the efficiency of multimodal data retrieval, and strengthening mechanisms that ensure consistent and bounded LLM-assisted reasoning. We also plan to address long-term process drift, practical integration with legacy manufacturing systems, and the development of assurance and certification pathways for safety-critical deployment. Together, these efforts will help transition the hybrid architecture from a validated research prototype to a robust and scalable industrial diagnostic platform.

## Figures and Tables

**Table 1:** Comparison Between Numerical and Multimodal FDD Approaches.

Aspect	Traditional Numerical FDD	Modern Multimodal FDD
Primary data types	Scalar or low-dimensional numerical signals (e.g., temperature, pressure, current)	Heterogeneous multimodal data, including numerical signals, images, audio, vibration, and time-series
Typical data sources	Sensors, Programmable Logic Controller (PLC) readings, SPC parameters	Wafer inspection images, wafer maps, inline metrology, equipment vibration, acoustic signals, logs
Representation	Fixed thresholds, residuals, statistical features	High-dimensional spatial–temporal patterns, spectral features, semantic representations
Fault sensitivity	Effective for abrupt and well-defined faults	Sensitive to subtle, early-stage, and pattern-based anomalies
Interpretability	High for simple rules and thresholds	Often lower due to complexity and data heterogeneity
Scalability to new fault types	Limited; requires manual rule updates	Higher potential but requires advanced representation and reasoning
Handling of non-stationarity	Weak under process drift or aging	Better observability but increased reasoning complexity
Computational methods	SPC, residual analysis, regression	Computer vision, signal processing, deep learning, multimodal fusion
Typical challenges	Rigid rules, poor adaptability	Data alignment, cross-modal reasoning, lack of causal grounding
Relevance in advanced FABs	Insufficient alone for leading-edge nodes	Essential for detecting complex processes and equipment anomalies

**Table 2:** Comparison of Pure LLM and DML-LLM hybrid architecture across key operational dimensions.

Dimension	Pure LLM	DML–LLM Hybrid Architecture
Knowledge Representation	Unstructured, linguistic, heuristic-based knowledge	Combination of causal temporal fuzzy logic (DML); Flexible semantic knowledge (LLM)
Data Type Handling	Strong at unstructured/semi-structured data (images, text, tables)	Integrates both structured data streams and unstructured modalities
Inference Consistency	Context-dependent, unstable outputs; poor reproducibility.	Logic-constrained reasoning with stable repeating results
Traceability and Governance	No explicit causal path or decision node trace	Full causal–temporal path traceable through DML nodes
Explainability	Natural language only, limited intermediate reasoning	Narrative structural reasoning layers for dual explanation
Implementation Effort	Fast deployment, prompt only	Requires logic modeling; supports long-term maintenance and scalability
Human–Machine Collaboration	Q&A interaction, passive assistance	Engineer-in-the-loop with second opinion advisory
Application Scenarios	Non-critical or exploratory analysis	High-reliability, traceability required diagnostic decision support

**Table 3:** Responsiveness metrics for the three evaluated methods (TTD and TTRC).

Method	TTD (h)	TTRC (h)
SPC/FDC	7.4	15.2
Pure LLM	3.1	8.9
DML–LLM Hybrid	1.2	4.7

**Table 4:** Diagnostic accuracy metrics for the three evaluated methods (precision, recall, F1 score).

Method	Precision	Recall	F1 Score
SPC/FDC	0.68	0.52	0.59
Pure LLM	0.72	0.78	0.75
DML–LLM Hybrid	0.85	0.81	0.83

**Table 5:** Governance and effort metrics for the three evaluated methods (provenance completeness and triage time).

Method	Provenance Completeness (%)	PIE Triage Time (Min/Event)
SPC/FDC	18	72
Pure LLM	42	39
DML–LLM Hybrid	96	18

## Data Availability

The data supporting the findings of this study are proprietary and subject to confidentiality agreements. Therefore, the data is not publicly available.

## Abbreviations

The following abbreviations are used in this manuscript:

AI	Artificial Intelligence
AOI	Automated Optical Inspection
CoT	Chain of Thought
DML	Dynamic Master Logic
DMLD	Dynamic Master Logic Diagram
DPR	Dense Passage Retrieval
DFM	Degree of Failure Membership
ETL	Extract, Transform, Load
FAB	Semiconductor Fabrication Facility
FDC	Fault Detection and Classification
FDD	Fault Detection and Diagnosis
FiD	Fusion-in-Decoder
FTA	Fault Tree Analysis
FMEA	Failure Mode and Effects Analysis
HITL	Human-in-the-Loop
KG	Knowledge Graph
LLM	Large Language Model
MCP	Model Context Protocol
MES	Manufacturing Execution System
ML	Machine Learning
MoE	Mixture-of-Experts
PIE	Process Integration Engineer
PLC	Programmable Logic Controller
QA	Quality Assurance
RAM	Reliability, Availability, and Maintainability
RAG	Retrieval-Augmented Generation
RCA	Root Cause Analysis
RUL	Remaining Useful Life
SEM	Scanning Electron Microscope
SLM	Small Language Model
SPC	Statistical Process Control
SOP	Standard Operating Procedure
ToT	Tree of Thoughts
TTD	Time to Detect
TTRC	Time to Root Cause
WAT	Wafer Acceptance Test

## Appendix A

Taxonomy of Process Integration–Oriented FDD Objectives and Supporting System Capabilities. This table summarizes a system-level taxonomy linking Process Integration FDD objectives to the functional capabilities required to support them across the full manufacturing life cycle. Objectives span design-time robustness and safety co-engineering, operational monitoring, early warning, diagnosis, and RCA, and logistical and governance-related goals, including predictive maintenance, remaining useful life (RUL) estimation, recovery, and compliance-aligned decision support. Capabilities are organized across acquisition, storage, analytics, monitoring, diagnosis, decision, action, and governance layers, and are associated with representative methods based on physical models, data-driven inference, knowledge graphs, retrieval mechanisms, and language model–assisted reasoning. Core capabilities essential to Process Integration FDD are denoted by ★, while optional or enabling capabilities are denoted by ◇.

**Table A1:** Taxonomy of Process Integration FDD objectives and capabilities used in the case study.

★ = Core Capability ◇ = Optional/Enabling Capability			GOAL: Process Integration Management																			
			Fault Detection and Diagnosis										Status Evaluation									
			Design				Operations			Logistic			QA/RAM				Facility			Compliance		
FUNCTIONAL: Capability to Support		References	Design-time Robustness	Sensing and Data Robustness	Security & Safety Co-Engineering	Model-based and Digital Twin	Monitoring and Early Warning	Diagnosis and Root Cause Analyses	Fault-tolerant Control & Mitigation	Knowledge and Decision Support	Maintenance and RUL	Recovery and Continuity	Safety and Security	Production Reliability and Quality	Process Reliability and Quality	Asset Health and Availability	Maintenance Readiness	Control Performance	Data and Model Integrity	Human Operations	Environment and Utilities	Compliance and Governance
Acquisition	Image	[41]		◇																		
	Physical measurement	[42]		★																★		
	Control data	[43]		★																		
	Logs and time	[44]		◇																		
	Sensor health	[45]		★																		
Storage	Time-series store	[46,47]		★							★											
	Data lake/ warehouse	[48]		◇							◇					◇				◇		
	Vector store	[47]		◇														◇				
	Knowledge base	[28,29,30,49]	◇					★		★		◇						◇	◇		◇	
	Asset graph	[49,50]	◇					◇		◇					◇							
	Versions and access	[51]				★						★				★		★			◇	
Integration Analytics	ETL and cleaning	[48]																★				
	Features	[48]				★					★											
	Stats	[46]	★					◇			◇			◇								
	ML	[42]	◇			◇					★											
	Anomaly	[46]					★							★								
	Uncertainty	[52]					◇											◇				
	Drift check	[53]	★				◇										★					
Monitoring	Digital twin	[54]	★			★									★							
	Observers and residuals	[55]	★			★	★							◇			★					
	Limits and trends	[56]					★							★			★			★		
	SPC charts	[56]		◇			◇							★			◇					
	Event stream	[57]			◇		★		◇				◇		◇					◇		
Diagnosis	Root cause	[58]						★						★								
	Causal graph	[59]						★						◇								
	Twin compare	[54]				★		◇														
	Health index	[10,24]						◇			★				★							
Decision	LLM assistant	[30,33,60]								★									★			
	Expert rules	[61]			★				★	★							◇					
	Schedule optimizer	[23]								◇	★				★	◇						
	Risk summary	[62]			◇					★			◇	◇						◇		
	Action card	[63]							◇	◇									◇			
Action	Work order	[64]									★	★			◇	★						
	Spares and repair	[37]									◇	★			★	★						
	Robots/automation	[36]							◇			◇										
	Controller fallback	[35]			★				★			◇	◇				★					
	Feedback loop	[37,55]							★													
Governance	Data governance	[48,51]	★															★			★	
	HITL, SLA and Audit	[61,63]			★								★					★	★		★	
	Safety	[61,63]			★								★								★	
	Cybersecurity	[61]			★								★								★	
	Redundancy	[61]			◇				★			★										
	Data/model security	[51,61]		★														★				
